# Detailed behavioral assessment promotes accurate diagnosis in patients with disorders of consciousness

**DOI:** 10.3389/fnhum.2015.00087

**Published:** 2015-03-04

**Authors:** Yael Gilutz, Avraham Lazary, Hana Karpin, Jean-Jacques Vatine, Tamar Misha, Hadassah Fortinsky, Haggai Sharon

**Affiliations:** ^1^Department of Occupational Therapy, Reuth Rehabilitation HospitalTel Aviv, Israel; ^2^Brain Injury Division, Reuth Rehabilitation HospitalTel Aviv, Israel; ^3^Outpatient and Research Division, Reuth Rehabilitation HospitalTel Aviv, Israel; ^4^Sackler School of Medicine, Tel Aviv UniversityTel Aviv Israel; ^5^Institute of Pain Medicine, Tel Aviv Sourasky Medical Center Tel Aviv, Israel; ^6^Center for Brain Functions, Tel Aviv Sourasky Medical Center Tel Aviv, Israel

**Keywords:** disorders of consciousness, behavioral assessments, vegetative state, minimally conscious state, brain injury

## Abstract

**Introduction**: Assessing the awareness level in patients with disorders of consciousness (DOC) is made on the basis of exhibited behaviors. However, since motor signs of awareness (i.e., non-reflex motor responses) can be very subtle, differentiating the vegetative from minimally conscious states (which is in itself not clear-cut) is often challenging. Even the careful clinician relying on standardized scales may arrive at a wrong diagnosis.

**Aim**: To report our experience in tackling this problem by using two in-house use assessment procedures developed at Reuth Rehabilitation Hospital, and demonstrate their clinical significance by reviewing two cases.

**Methods**: (1) Reuth DOC Response Assessment (RDOC-RA) –administered in addition to the standardized tools, and emphasizes the importance of assessing a wide range of motor responses. In our experience, in some patients the only evidence for awareness may be a private specific movement that is not assessed by standard assessment tools. (2) Reuth DOC Periodic Intervention Model (RDOC-PIM) – current literature regarding assessment and diagnosis in DOC refers mostly to the acute phase of up to 1 year post injury. However, we have found major changes in responsiveness occurring 1 year or more post-injury in many patients. Therefore, we conduct periodic assessments at predetermined times points to ensure patients are not misdiagnosed or neurological changes overlooked.

**Results**: In the first case the RDOC-RA promoted a more accurate diagnosis than that based on standardized scales alone. The second case shows how the RDOC-PIM allowed us to recognize late recovery and promoted reinstatement of treatment with good results.

**Conclusion**: Adding a detailed periodic assessment of DOC patients to existing scales can yield critical information, promoting better diagnosis, treatment, and clinical outcomes. We discuss the implications of this observation for the future development and validation of assessment tools in DOC patients.

## INTRODUCTION

Disorders of consciousness (DOC) following extensive acute brain injury (either hypoxic/ischemic or traumatic) are some of the most enigmatic neurological syndromes described. Since the first description of the so called “Vegetative State Syndrome” by Jennett and Plum (1972), there have been great advances in the nosology of chronic DOC, varying from coma to vegetative and minimal conscious states, with names and criteria still evolving. The current diagnostic criteria for vegetative state (VS) were formalized by a 1994 US Task Force of the American Academy of Neurology and a second task force published consensus recommendations defining this new clinical entity termed “minimally conscious state (MCS)” in Giacino et al. (2002). The operational criteria for minimally conscious state thus separated non-communicative vegetative patients from non-communicative patients showing subtle and fluctuating behavioral signs suggesting awareness. Emergence from the minimally conscious state was defined by overt functional communication or functional use of objects (“emerging MCS”). These dichotomic diagnostic categories were only lately challenged when a further sub-grouping of the MCS was suggested (Bruno et al., 2009) and on 2011 published in ICD9 (WHO) as (Charland and Laureys, 2012): (1) MCS- Non-communicative patients with severely impaired responsiveness showing inconsistent but definite signs of consciousness; (2) MCS-Minus – Minimal levels of behavioral interaction characterized by the presence of very basic non-reflex movements (e.g., orientation of noxious stimuli, pursuit eye movements that occur appropriately in relation to relevant environmental stimuli); and (3) MCS-Plus – the presence of overt command following, intelligible verbalization or reproducible gestural or verbal yes/no responses. The upper boundary of MCS is defined by the recovery of functional communication or functional use of objects (Giacino et al., 2002).

Clinical assessment of persons with DOC therefore still relies on observing behavioral responses to stimuli and drawing inferences about the underlying state of consciousness ( Laureys et al., 2005; Giacino and Smart, 2007; Seel et al., 2010). However, many clinicians still encounter difficulties in correctly diagnosing patients who show only trace signs of fluctuating awareness. Many low responders are, therefore, still misdiagnosed as being vegetative. Estimates of misdiagnosis among patients with DOC have been very high, ranging from 37 to 43% in some studies (Childs et al., 1993; Andrews et al., 1996; Schnakers et al., 2009). A sensitive standardized neurobehavioral assessment scale may thus help decrease diagnostic error and limit diagnostic uncertainty (Schnakers et al., 2009).

Accordingly, specialized and standardized assessment tools designed for use in patients with DOC were first introduced in rehabilitation settings in the early 1990s (Kalmar and Giacino, 2005) and have multiplied since then (Seel et al., 2010). Many scoring systems have been developed and validated for the quantification and standardization of the assessment of consciousness (Majerus et al., 2005). However, detection of behavioral signs of consciousness is nevertheless subject to inter-rater variability and is often confounded by unpredictable fluctuations in arousal, underlying sensorimotor impairment, unrecognized cognitive and language deficits, and sedative medications. Even when there is agreement about the behavior observed, there may be assessor variability when inferring consciousness (i.e., differentiating between reflexive or voluntary movement; Schiff, 2006; Giacino and Smart, 2007; Seel et al., 2010).

A major drive to accurately define the precise level of consciousness is to better define the prognosis. While determining the prognosis of a specific DOC patient remains extremely challenging, there is clearly a better prognosis for recovery from the MCS than VS. Luauté et al. (2010) have recently reported that a high percentage (33%) of patients diagnosed as MCS at 1 year following a brain insult improved during a 5-year follow-up period, compared with none diagnosed as being in the VS (0%). As for the possible timeline of neurological improvement, while many individuals recover consciousness quickly, some rare cases may display impaired consciousness for prolonged periods before recovering (Nakase-Richardson et al., 2012). The, 1994 US Task Force emphasized that the term “permanent VS,” defined as 12 months after traumatic brain injuries (TBI) and beyond 3 months after non-traumatic injuries, refers to prognosis and claimed it as the point after which recovery of consciousness is ‘highly improbable’ but not impossible (Multi-Society Task Force on PVS, 1994; Royal College of Physicians, 2013). According to early epidemiologic studies on prognosis of VS, late recovery of consciousness, is very unlikely (Estraneo et al., 2010). Nevertheless, in both VS and MCS there are some isolated reports of recovery even after many years (McMillan and Herbert, 2004; Voss et al., 2006; Fins et al., 2007). In light of such documented late recoveries, it is crucial to view the temporal definitions as probabilities. Estraneo et al. (2010), who documented late recovery of responsiveness and consciousness in 12 out of 50 VS patients (12%), further demonstrated the necessity of long-term monitoring in patients with chronic DOC.

Another possible reason to pursue a detailed diagnosis of the level of residual awareness and its behavioral attributes is to try and tailor the best appropriate care for the patient, since there seems to be a distinct difference in awareness between different subgroups of DOC patients, but these may be dynamic and present in a variety of subtle ways. This is in line with the approach (as also presented for example, by Liberati et al. (2014) in this issue of Frontiers (Liberati et al., 2014), that it may prove more beneficial to categorize patients based on multiple detailed ordinal behavioral scores and diversified assessment procedures rather than relying on rather simplistic VS and MCS dichotomic diagnostic categories.

## BACKGROUND

One of the roles of Occupational Therapists who work with people with DOC is to recognize patient responses for the purpose of assessment and treatment. The Coma Recovery Scale-Revised (CRS-R) is the most commonly used behavioral scale for this purpose (Lancioni et al., 2014). It has a standardized rating scale that relies on consistent administration and scoring criteria (Giacino and Smart, 2007). The CRS-R is particularly helpful in discriminating between the VS and MCS (Majerus et al., 2005). During the years of work at “Reuth Rehabilitation Hospital” two main dilemmas have arisen in the work with the DOC population. The first dilemma is that some patients, according to standardized assessments, have shown no purposeful responses or low-level responses and yet the staff have the impression that the level of consciousness is higher. The second dilemma is how to periodically determine how long to continue rehabilitative treatment.

Regarding the first dilemma, there are several possible reasons for the discrepancy between the scores on standardized tests and the impressions of the staff:

1. Responses are present (albeit maybe not consistently) but there is difficulty recognizing them in patients who suffer from multiple sensory, motor, and cognitive impairments (Zasler et al., 1994).
2. Responses are present but eliciting them requires intense encouragement and work on the part of the therapist.
3. Responses are present but they do not meet the criteria set out by standardized assessments (verbal commands given only once, response time is limited- whereas these patients may require repeated commands to elicit responses or responses appear after several seconds (Majerus et al., 2005).
4. Patients with DOC exhibit a wide range of possible behaviors (Multi-Society Task Force on PVS, 1994; Royal College of Physicians Working Group, 1996), which are not represented in items listed on standardized scoring systems. As such, many patients may display idiosyncratic behaviors as a marker for awareness that is patient-specific.

In order to deal with this challenge, and in an effort to recognize more complex responses, the Individualized Quantitative Behavioral Assessment (IQBA) technique is used in addition to the CRS-R. IQBA techniques represent a second means of objectively investigating behavior in patients with DOCs. IQBA is a useful adjunct to standardized assessment strategies and is an effective means of assessing command-following ability when behavioral responses are ambiguous, but is underutilized in the clinical setting (Giacino and Smart, 2007).

At “Reuth Rehabilitation Hospital,” over the course of several years, many case studies were collected in order to help develop a comprehensive assessment tool called the Reuth Disorders of Consciousness Response Assessment (RDOC-RA) in order to recognize a variety of high-level responses.

This tool includes detailed information and explicit instructions that are meant to broaden and enhance the clinician’s repertoire for assessing and treating DOC patients. It is built as a list that includes all body parts and the different movement possibilities of each body part. In addition, the tool details the different possibilities for mediation in order to help the patient to elicit responses and thereby help to determine the patient’s specific level of consciousness. The tool is not a diagnostic tool that stands alone, since it has not yet been properly validated and is an in-house procedure, but rather a supplement to the existing standardized tools that can be used to improve the assessment process, especially the assessment of command following, and allow the patient different possibilities to indicate their responses.The RDOC-RA is made up of two parts:

1. Assessment tool- DOC Movement Checklist
2. Documentation tool- DOC Response Profile

1. The DOC Movement Checklist is a detailed list of all body parts and the different movements of each body part. The goal is to carry out a detailed search of command-following assessment for any movement/response, whether spontaneous or not, in a given patient. All present movements are then assessed to determine whether they can be used with the patient’s control as a means of communication. The search for voluntary movements is done through the use of different cues in order to elicit the responses (e.g., tactile or kinesthetic cues). Although this population may have many sensory, motor and cognitive deficits, the purpose of the cues is to assist in eliciting the volitional responses despite the deficits.
2. The DOC Response Profile is a more detailed documentation of the specific response profile of the patient. It has explicit instructions on which types of commands can be used to elicit responses as well as documentation of whether there were enhancing or impeding elements that helped or hindered the elicitation of responses. The tool allows the clinician to record response time as well as the consistency of responses within each treatment session (how many times a patient was able to perform them). In addition, the tool allows the assessor to determine if the response can be used for communication and in which manner. Through the use of detailed documentation subtle changes can be monitored over time to track the dynamics in responses. Once documented, information can be shared with family members and staff, who can continue to practice and/or communicate with the patient using the elicited responses.

The RDOC-RA comes with instructions on how to fill out the DOC Movement Checklist for each response observed. The use of the DOC Response Assessment assists in recognizing higher level responses, thereby preventing misdiagnosis of the patient’s level of consciousness, which can influence decisions regarding further intervention.

**FIGURE 1:**
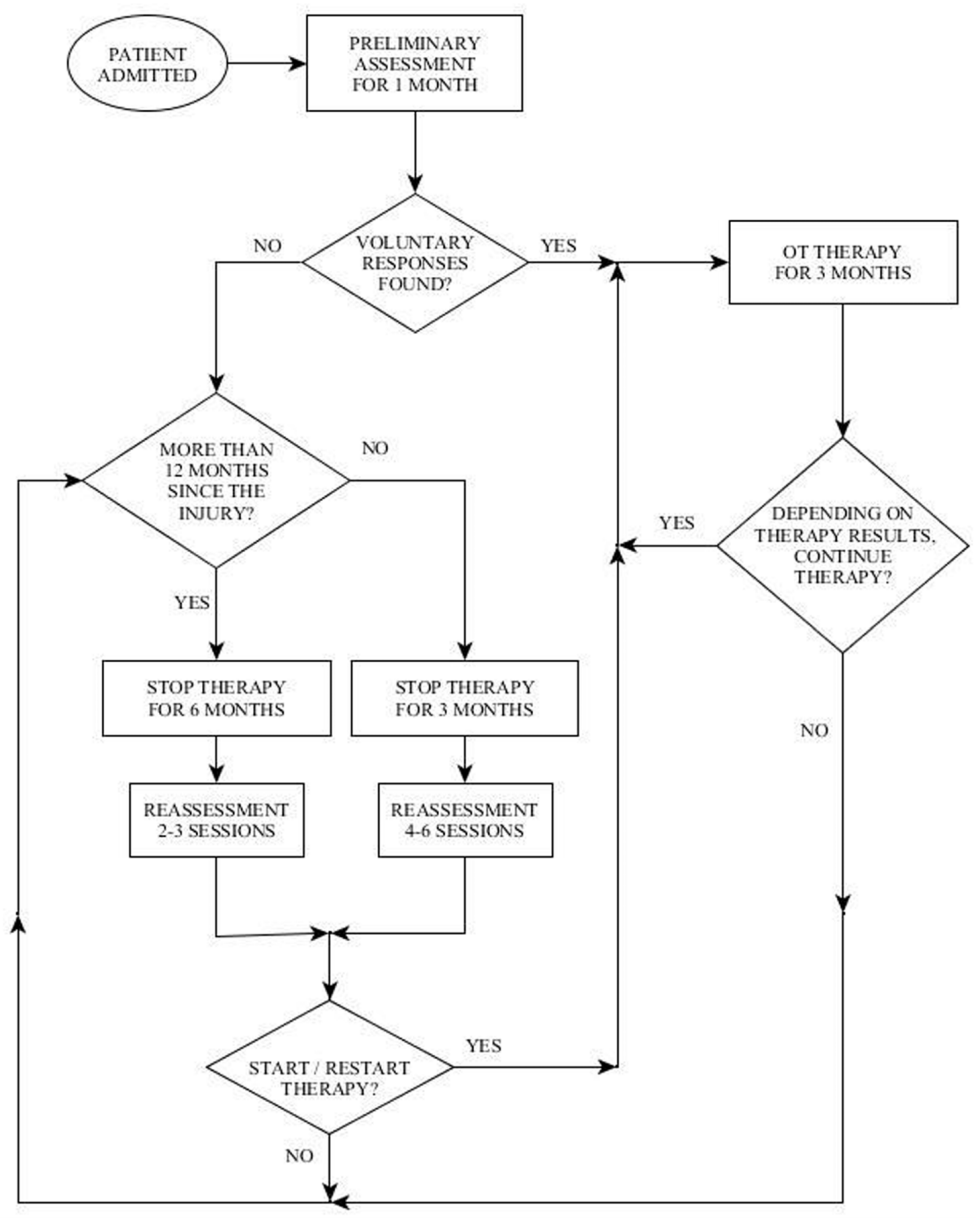
**Depiction of the Reuth DOC Periodic Intervention Model (RDOC-PIM)**.

The second challenge mentioned above in working with the population of DOC, is determining how long to continue treatment, the frequency of treatment and when to discontinue treatment. These questions arise due to the fact that this population is characterized by an inconsistency of responses and fluctuating status (Giacino et al., 2009). In order to cope with this challenge the Reuth DOC Periodic Intervention Model (RDOC-PIM) was created at “Reuth Rehabilitation Hospital.” This model dictates defined times to perform periodic assessments of the patient during the rehabilitation process (**Figure 1**). The clinician then uses clinical judgment regarding the continuation, cessation, or reinstitution of treatment based on 3 factors: (1) the quality of the response (high level/low level), (2) the consistency of the response/s: how often it appears during a treatment session, and whether the responses are present in each/most/some treatment sessions, (3) dynamics: is there a change over time in the number of responses, the level of responses or the consistency of responses.

In this paper, we present two case studies to illustrate the use of the RDOC-RA and the RDOC-PIM. The first case study demonstrates the added benefit from using the RDOC-RA as an adjunct to the CRS-R by providing the ability to recognize responses that have functional application for the use of yes/no communication with the patient. The second case study demonstrates the importance of the RDOC-PIM in identifying changes over time in the responses of a patient and identifying the rehabilitation potential of the patient even a year post injury.

## CASE STUDY 1– REUTH DOC RESPONSE ASSESSMENT AS AN ADJUNCT TO THE CRS-R

AC, a 38 years old male, sustained severe anoxic brain damage due to respiratory arrest following acute pancreatitis. After stabilization of his acute condition he was initially diagnosed as being in a vegetative state. AC was admitted to “Reuth Rehabilitation Hospital” on October 2010. He underwent an occupational therapy (OT) assessment and intervention began immediately following his admission. No spontaneous movements were observed other than blinking. The ongoing OT assessment included, among other things, CRS-R and RDOC-RA: the first performed 1 month post admission to “Reuth” (6 months post injury) and the second 3 months after intervention (9 months post-injury).

According to the CRS-R administered at both assessments AC was diagnosed as MCS minus, as determined according to his performance of visual pursuit (**Table 1**). The DOC Movement Checklist below (**Table 2**) summarizes the OT assessment process at the same two points in time as the above.

**Table 1:** Patient’s AC score on the first and second administration of the CRS-R.

CRS-R subscales	Months post injury	
	6 months (10/2011)	9 months (01/2012)
Auditory	1	1
Visual	3	3
Motor	2	2
Oral motor/verbal	1	1
Communication	0	0
Arousal	1	1
Total score	8/23	8/23

**Table 2:** Patient AC’s movements on the first and second administration of the DOC movement checklist.

	Date	10/2011	11/2011–01/2012	
**1**	**Head**		
	Right	+	+
	Up	+	+
	Up	-	
	Down	-	
	Closes eyes	-	
	Double blink	+	+
	Prolonged blink	-	-
	Winks		-
	**Gaze**		
	To the right		-
	To the left		-
	**Hands**		
	**Thumb**	**L/R**	**L/R**
	Flexion	-/-	/
	Extension	-/-	/
	**Index finger**	**L/R**	**L/R**
	Flexion	-/-	-/-
	Extension	-/-	-/-
	**Elbow**	**L/R**	**L/R**
	Flexion	-/+	/+ **supp
	Extension	+/-	/+ **supp

**According to DOC Response Assessment Guidelines indicates yes/no communication.

At the first assessment point, AC was found to be able to follow commands to move his head to both sides and to blink twice. At the second assessment he could move his right elbow active-assisted and by flexing/extending his elbow he was able to communicate yes/no answers which allowed for establishing communication. A qualitative documentation of his responses using the RDOC Response Profile can be found in **Table 3**.

**Table 3:** Patient AC’s responses profile on the first and second administration of the DOC response profile

Date	Movement	Response profile
10/20101 month longassessment uponadmission (6 monthspost injury)	Turns head right and left, double blink	Instructions: verbal, oralCommunication: noneEnhancing conditions:Instructions repeated 5–6 times.Quiet environmentRestricting conditions:—Response time: 40–50 s from initial instructionConsistency:During one session: 2–3 timesAfter 10–15 min from the beginning of treatment a noticeable decline in responses.During intervention period: responses noted in most treatment sessions.Notes: responses performed slowly.
11/2010–01/2011summary after 3 months of intervention (7–9 months post injury)	Turns head right and left, double blink	Instructions: verbal, oralCommunication: noneEnhancing conditions:Instructions repeated 3–5 times.Quiet environment.Encouraging tone of voice.Presence of family memberRestricting conditions:—Response time: 20–30 s from initial instruction.Consistency:During one session: 4–5 times for each response. After 20 min of treatment a marked decline in ability to respond.During intervention period: Responses seen during most treatment sessions.Notes: responses performed slowly.
	Elbow movement**Extension = yesFlexion = no	Instructions: verbal–oral and active assisted (support to elbow)Communication: present. Flex = no, extend = yes. Answers autobiographical questions, general knowledge questions,Enhancing conditions:Instructions repeated 2–3 times.Restricting Conditions: noneResponse time: 10 s from initial instruction.Consistency:During one session: answers 5–7 questions/session. After 20 min a marked decrease in ability to respond.During intervention period: Responses seen in most sessions.Notes:Responses performed slowly.Elbow responses were practiced 2–3 times as idiosyncratic gesture before becoming reliable yes/no communication.AC required rest for 30–60 s after 5–6 min of therapy.After the rest AC required a reminder and further practice to perform the yes/no communication sign

**According to DOC Response Assessment Guidelines indicates yes/no communication.

Using the RDOC Response Assessment (comprising these two procedures) it was demonstrated that AC could follow a command as early as the first month of his admission to the rehabilitation facility and therefore fell into the MCS plus subcategory. During the second phase of assessment AC was able to communicate using supported elbow movements that indicated yes/no and answer biographical and general knowledge questions using those movements, although inconsistently. This denotes emergence from MCS.

Therefore, there was a crucial difference between the patient’s level of consciousness according to the CRS-R (MCS- at both time points) compared to the RDOC Response Assessment (MCS^+^ at 1 month and emergent MCS at 3 months). According to the RDOC Response Assessment, AC was able to communicate with his environment and answers questions using his yes/no communication sign, an action indicating a higher level of cognitive function. These functions are not highlighted by the CRS-R. The method of following commands is not fulfilled by the criteria set by the CRS-R that requires consistency of 4/4 times, response time <less than 10 s and AC was not able to answer the questions on the communication subscale set out by the manual. In the DOC Response Profile there is a detailed description of responses that can serve as a method to practice communication between the patient and staff or family members.

## CASE STUDY 2 – REUTH DOC PERIODIC INTERVENTION MODEL

BR, a previously healthy 33 year old male, suffered a severe TBI due to fall from an altitude of 8 m in August 2011. He had a large right subdural hematoma, which was surgically evacuated, and diffuse brain injury. After he developed hydrocephalus 2 months later, a permanent ventriculo- peritoneal shunt was installed. He was admitted to “Reuth Rehabilitation Hospital” in January 2012 (i.e., 4 months after the injury) with the diagnosis of VS.

Upon admission, the OT intervention process began and was continued for the following month. During this time the patient displayed consistent gaze preference to the right and no spontaneous active movements were noted. Accordingly, during the assessment process it was noted that he displayed reduced reactivity when the therapist stood to his left, so in order to improve cooperation and promote eliciting reactions, the therapist made it a point to stand to his right side. During the initial phase of the 1 month assessment with DOC Movement Checklist BR’s range of motor behavior was carefully documented (see **Table 4**). Using the DOC Response Profile assessment the impression was that these movements, such as opening the mouth and closing the eyes, could be elicited voluntarily by the patient, though inconsistently (see **Table 5**).

**Table 4:** BR’s movements on the DOC Movement Checklist.

	Movement/date	01/2012 5 m post injury	02/2012–04/2012 6–8 m post injury	08/2012 12 m post injury	03/2013 9 m post injury
**1**	**Head**					
	Right	-	-				
	Left	-	-			
	Up					
	Down	-				
**2**	**Eyes**				
	Closes eyes	+	+	+	+		
	Double blink	-	-				
	Quick blink	-	-			
	Prolonged blink	+	+	+	+	
**3**	**Gaze**				
	To the right					
	To the left	-	+	+			
	Down		-			
	Upon presentation of two objects held in the **vertical** plane, is able to gaze at one of them, on command		**	**	+		
**5**	**Mouth**				
	Open	+	-			
	Close	-				
	Smile				+	
**Upper extremities**
**8**	**Elbow**	**L/R**	**L/R**	**L/R**	**L/R**
	Flexion	-/-	/	/	/
	Extension	-/-	/	/	/		
**10**	**Hands**					
	**Thumb**	**L/R**	**L/R**	**L/R**	**L/R**	
	Flexion		/	/	/+**	
	Extension		/	/	/+**	
	**Index finger**		**L/R**	**L/R**	**L/R**	
	Flexion		/	/+ KNST	/+**		
	Extension		/	/+	/+**	
**Whole hand**
	**Hand shake**	**L/R**	**L/R**	**L/R**	**L/R**	
	Once	-	/+	/+	/+	
	Double		/+	/+	/+	
	Wave “hello/goodbye”		/+ IMIT	+/ IMIT	/+	
	Grasps an object		/	/	/+

**According to DOC Response Assessment Guidelines indicates yes/no communication.

**Table 5:** BR’s response scores on the DOC Response Profile.

Date: 01/2012, 1 month assessment, 5 months post injury
**Movement/s**	**Response profile**
Close his eyes, prolonged closing his eyes, open his mouth with a minimal range of motion	Command: verbal, oral Communication: pre-designated sign of prolonged closed eyes to signal “yes”- unclear (change in blinking pace), inconsistent.Enhancing conditions:Therapist stands on patient’s right side Restricting Conditions:—Response time: 10–30 s from initial command Consistency:- During one session: 2–4 times for each response After 5–15 min from the beginning of treatment B.R. stopped responding/closed his eyes- During intervention period: Responses noted every 2–3 sessionsNotes: —
∙ OT intervention was continued for the next 3 months – see text.
**Date: 02-04/2012, 3 months intervention, 6–8 months post injury**
Close his eyes, prolonged closing his eyes, move gaze upward and downward (in the right field of vision)**, single and double handshake with right hand, waves “goodbye” with fingers of right hand.	Command: verbal, oral. Waves “goodbye” – verbal + imitation.Communication: pre-designated sign of prolonged closed eyes to signal “yes”- still unclear (change in blinking pace) – non functional.Able to use up/down gaze towards cards presented in right visual field in a vertical manner with yes/no written on them.Enhancing conditions:Therapist stands on patient’s right sideRestricting conditions:—Response time: 10–30 s from initial commandConsistency:- During one session: 2–4 times for each responseAfter 5–15 minutes from the beginning of treatment B.R. stopped responding/closed his eyes- During intervention period: Responses noted every 2–3 sessions.Notes: Doesn’t follow command to open his mouth anymore, all responses noted were present since the two first weeks of the intervention process and there was no change in the consistency of the range.
∙ OT intervention was discontinued for the next 3 months – see text.
**Date: 08/2012, 1 month re-assessment, 12 months post injury**
Close his eyes, prolonged closing his eyes, move gaze upward and downward (in the right field of vision)**, single and double handshake with right hand	Command: verbal, oral. Flex/extend right index – verbal + kinesthetic Communication: lower frequency of use of up/down gaze towards yes/no cards for answering questions (1–2 questions during session).Enhancing conditions:Therapist stands on patient’s right side Restricting conditions:Warm room temperature Response time: 10–30 s from initial command Consistency:- During one session: 2–4 times for each response After 5–15 min from the beginning of treatment B stopped responding/closed his eyes- During intervention period: Responses noted every 2–3 sessions.Notes: Sometimes BR smiled appropriately to the situation but was unable to smile in response to a command.
∙ OT intervention was suspended for another 6 months – see text.
**Date: 03/2013, 1 month re-assessment, 19 months post injury**
Close his eyes, prolonged closing his eyes, move gaze upward and downward, single and double handshake with right hand, waves ”goodbye” with fingers of right hand,flex/extend right index,flex/extend right thumb, holds object when placed between his right index finger and thumb (e.g., a key, pencil), smile, able to draw a line – vertical, 4–5 millimeters long**	Command: verbal, oralCommunication: use of up/down gaze towards yes/no cards for answering questions – non functional.Use of line drawing to signal “yes,” answers 6–8 general and biographical questionsEnhancing conditions:—Restricting conditions:Warm surrounding temperatureResponse time: 5–15 s from initial commandConsistency:- During one session: 5–6 times for each responseAfter about 10–15 min from the beginning of treatment B closes his eyes, able to reopen his eyes and respond after talking to him about his philosophy, arts, or telling jokes- During intervention period: BR showed responses during most of the assessment sessions.Notes: BR smiled appropriately for the situation and inconsistently was able to smile in response to a command.
∙ OT intervention was reinstated and is still ongoing – see text.

**According to DOC Response Assessment Guidelines indicates yes/no communication.

Therefore, according to the DOC Periodic Intervention Model (RDOC-PIM, **Figure 1**), in light of the impression of voluntary responses displayed by BR, it was decided to continue with OT intervention for the next 3 months.

Three months later (6–8 months following the injury) he displayed a greater variety of motor responses (though still inconsistently) when he was able to close his eyes for a prolonged period on demand, move his gaze upward and downward, and perform a single and double handshake with his right hand (**Table 5**). According to the RDOC-PIM, although high-level responses were noted and there was communication ability, the consistency of the responses in the therapy sessions was low and with no dynamics and therefore it was decided to stop OT therapy at this time for a period of 3 months. Before stopping therapy, however, the family was given guidance on how to communicate with BR using the yes/no cards.

Three months later he was reassessed once more for a 1 month period, but displayed no change in his responsiveness in terms of range or consistency (**Table 5**). According to the RDOC-PIM, in light of the fact that the time since initial injury was already 1 year, OT intervention was stopped for a period of 6 months. Following this period, over 19 months after the injury, he was again reassessed for a period of 1 month. This time he displayed new responses – he was able to hold an object when placed between his right index finger and thumb (e.g., a key, pencil), he smiled intermittently when spoken to, and was able to draw a vertical, 4–5 millimeters long line. According to the RDOC-PIM, in light of new high-level responses and the ability to communicate more consistently, therapy was immediately resumed.

**FIGURE 2:**
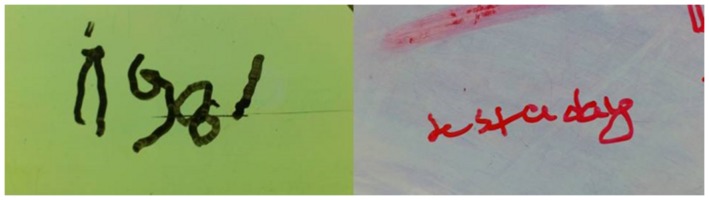
**Drawings made by BR during his OT sessions, as described in the text.** Note the answer to the question “when is your date of birth?” (answer: “1981,” on the left), and the response when asked what is a favorite song of his (answer: “Yesterday” by the Beatles, on the right).

Over the course of the following months, BR continued to slowly progress and a few months later emerged from the minimally conscious state, with initial signs of neurological improvement first manifesting during the course of intense OT therapy. He was able to use yes/no communication by closing his eyes, use a switch with his right hand and even draw and write single words for the first time during OT therapy (**Figure 2**).

He still continues his OT therapy on a regular basis, with improving communication and cognitive skills.

## DISCUSSION

Correct diagnosis in patients with DOC is of paramount importance. Apart from the ethical and psychological implications of a misdiagnosis for the patient and the family, correct diagnosis may better patient care and allow us to develop further therapeutic interventions that are patient centered and tailored (Liberati et al., 2014). Of note, the attempt at delineating separate diagnostic categories based on a constantly evolving concept of these syndromes, while advancing our phenomenological categorization of these patients and providing important diagnostic information can often be the source of much confusion. For example, some patients who are classified as “permanent” VS can in fact emerge into MCS and continue to improve. In addition, clinical diagnosis in current medical practice is still hampered by numerous assessor-dependent variables, further confounding the effort to classify patients into rigid categories. Moreover, standardized testing may easily overlook the unusual phenomenological manifestations of covert awareness in individuals with severe neurological injuries. More advanced diagnostic methods, such as electroencephalography (EEG), transcranial magnetic stimulation (TMS), EEG-TMS combination and functional magnetic resonance imaging (fMRI) have shown great promise while revolutionizing our understanding of the possible residual brain capabilities in these patients, both cognitive (Boly, 2011) and emotional (Sharon et al., 2013) but remain investigational and seem to still be a long way from being incorporated into clinical medical practice (Boly, 2011).

The purpose of this article was to present our approach, that an extensive and periodic patient assessment is required in DOC patients alongside standard assessment tools in order to recognize subtle high level responses. To support this notion we present as an example two detailed clinical procedures (a detailed assessment tool and a detailed follow up algorithm) that were developed for in-house use at the OT department in “Reuth Rehabilitation Hospital.” By using them we try and minimize the risk inherent in inferring cognition and emotion from a limited range of possible behaviors, as are assessed using standardized scores only.

The case studies we presented illustrate the clinical use of these two procedures. In the first case study, the use of the RDOC-RA demonstrated how the patient’s responses were methodically probed and recognized. Crucially, these responses were not uncovered using the standardized assessment tool. Therefore, in this patient the use of the detailed RDOC-RA lead to a more accurate diagnosis of the level of consciousness and awareness and even allowed us to form a system that allowed for the establishment of a yes/no communication method.

The second case study showed how the follow up algorithm allowed for the careful repeated assessments of the patient’s neurological condition at predetermined time intervals along the way. This approach resulted in the identification of a rehabilitative potential at the slightest sign of a change in the neurological status, eventually leading to the recognition of consistent patient responses and the reinstatement of continuous treatment.

Of note, the tools that we presented here have not been validated – they should be reproduced in large series and by different assessors under different clinical conditions assessing different patient groups in order to be considered valid and recommended. However, while in no way a stand-alone replacement to existing standardized tools, they serve to raise the issue of how a detailed, on-going and patient-tailored clinical assessment may prove crucial in the diagnosis and delineation of treatment in DOC patients.

Standardized tools are of crucial importance in the assessment of patients with DOC. Extensively validated tools such as the CRS-R are indispensable in creating an academic atmosphere that uses standard nosology and assessment, aim to minimize observer bias, and delineate the different groups of DOC patients. They also serve the clinician well in minimizing errors and in communicating with family and caregivers. However, we know so little about these complex disorders that reliance on a predetermined and relatively small number of standard responses as a sign of awareness may cause the assessor to unwillingly ignore other, more personalized, ways to communicate with a specific patient. In other words, while standardization is mandatory in order to better formulate our research questions and clinical guidelines, one must always keep in mind that in a complex brain disorder the personal neurological profile in a given patient may yield unconventional phenomenological evidence of awareness (Zasler et al., 1994), and not only those depicted in standardized and validated procedures (Bernat, 2006).

The management of DOC patients raises complex practical and ethical dilemmas. Clinicians working in this area often struggle with the questions of whether all possibilities of finding responses and realizing the rehabilitative potential of the patient were exhausted and how to assess the cases of late recovery. To that important aim, we feel that an exhaustive periodic clinical assessment and documentation of all the patient’s range of behavioral output may yield valuable information to complement that which is acquired using standardized assessment tools.

## Conflict of Interest Statement

The authors declare that the research was conducted in the absence of any commercial or financial relationships that could be construed as a potential conflict of interest.
