# Supplementary material for: Detailed behavioral assessment promotes accurate diagnosis in patients with disorders of consciousness
Source: Front Hum Neurosci. 2015 Mar 4;9:87. doi: 10.3389/fnhum.2015.00087 (PMC4349183; doi:10.3389/fnhum.2015.00087)

Supplementary Appendix

For the manuscript: "Detailed Behavioral Assessment Promotes Accurate Diagnosis in Patients with Disorders of Consciousness"

Yael Gilutz, Avraham Lazary, Hana Karpin, Jean- Jacques Vatine, Tamar Misha , Hadassah Fortinsky and Haggai Sharon

This document includes:

**Appendix A** - General approach to the Reuth DOC Response Assessment

**Appendix B** – Administering the Reuth DOC Response Assessment

1. DOC Movement Checklist
2. DOC Response Profile

**Appendix C –** Guidelines for the use of the Reuth DOC Periodic Intervention Model

**Appendix A: General approach to the Reuth DOC Response Assessment**

1. Gathering Information

Information should be gathered from close family members/friends regarding personal details and past interests. This will help in:

1. Stimulating the patient if he closes his eyes
2. Encouraging him to participate
3. Creating questions that will serve for practicing yes/no communication.

Information can consist of biographical details, information about family members, areas of interest, likes and dislikes, experiences and past adventures.

1. Optimal Timing for gathering information
2. Family and staff members should be questioned to help determine when the patient is most aware and aroused.
3. The assessment should be done during different times of the day to determine best possible outcome.
4. The influence of other activities should be determined to allow the best possible responses – E.g. Does prolonged sitting or other treatment cause fatigue? Does treatment that focus on physical activity Do motor or physical treatments or sensory stimulation help to arouse the patient?

It is best to perform the assessment when the patient is not too fatigued and when they are most aware and aroused. The overall time frame for the evaluation is 30 minutes.

1. Positioning the Patient

It is preferable to assess the patient in a variety of positions to determine the optimal position to elicit responses. It is known that a vertical position has good influences on arousal but it must be weighed against the fact that sitting may be too fatiguing for the patient with DOC. In addition, for the patient with limited mobility, sitting may further reduce the already limited range of motion and therefore impede the ability to perform motor responses.

1. The presence of family members and /or other staff during the assessment

Try to assess if the patient's responses are stronger in the presence of family members or other staff. If so, it is suggested that family members be present and say the instructions for the assessment with the guidance of the therapist/assessor.

1. Arousal Protocol

At the beginning of the assessment, if the patient's eyes are closed or if they close during the assessment, the patient should try to be stimulated by calling their name, a light touch to their face or body, sensory stimulation, non- painful passive movement of the limbs or talking about areas of interest of the patient.

Pain or shock should not be used as a stimulant for arousal if possible. These patients are quite helpless, passive and dependent on their surroundings. Many times there are sensory deficits, such as vision, hearing or sensation impairments, that further enhances their sense of dependence and lack of control of their surroundings. It is important, therefore, when assessing and treating this population, to do it in a pleasant manner including using words of encouragement, persuasion and reinforcement and to encourage active participation.

1. Giving Instructions

Instructions should be given clearly, succinctly and repeated several times.

After repeating the instructions several times, wait for a response, which sometimes may take up to 2 minutes.

Responses are sometimes enhanced when there is a change in tone of voice, (e.g. calmer, more excited, monotone).

If no response is noted, or only a partial response, use the protocol of the DOC Movement Checklist for giving instructions.

1. Specificity of Instructions

Care should be given in the words chosen for instructions to ensure that the patient is performing the responses required by the instructions and not using a pre-designated sign. For that reason, questions should be short not including a sign as part of the instructions for a desired response. (E.g. "If you want to leave the room, close your eyes." In this case the patient may perform the instructions at the end of the sentence and seen as a response to the question.) Try to differentiate between the question and the response that answers the question. First try to establish the pre-designated sign and then ask the question. E.g. "Do you want to leave the room?" or "Show me with your eyes/hand if you want to leave the room."

1. When a different response appears to the one requested
   If a different response appears to the one requested, ask the patient to repeat the response in order to determine if it is an active, willed response or reflex.
   Different responses may be: in another area of the limb requested to perform the response, looking at the limb requested to perform the response, use of
   another body part to perform a response.
2. Full Free movement

In addition to giving instructions to perform defined movements/responses as outlined in the DOC Movement Checklist table, the patient should be asked to move each body part as fully as he can.

1. Mass movement as a response

If the patient performs a response that is not specific, but rather a mass movement of a body part/s due to severe neurological impairment that has affected motor control, ask the patient to repeat the movement to determine if it is an active, willed response or reflex.

Though mass movement can often be understood as a reflex, it should be further investigated if it can be used actively by the patient as a pre-designated sign or as a purposeful movement for using assistive technology.

1. Stereotypical Movements

If stereotypical movements are seen as a response to instructions, the patient should be asked to stop the response or to change the pace of the response, in order to determine if it is an active, willed response. Requests to stop or change the pace of stereotypical responses can also be suggested if the response appears spontaneously without an instruction to perform a desired movement.

1. Assisted –Active responses

When an assisted-active response is performed to indicate yes/no communication, care should be given by the assessor not to redirect the patient to perform the required response.

In order to avoid this, family members or close friends can ask questions that the therapist does not know the answers to, in order to determine the authenticity of the direction of the response, without the subconscious help of the therapist.

1. Pre-Designated sign or yes/no communication

Any response that the patient performs reliably should be assessed with an eye to its potential use as a pre-designated sign or as an active movement for using assistive-technology.

Even if the responsive movement is not performed consistently, it should be assessed whether when using it for communication enhances its consistency.

1. Finding 2 reliable responses

If possible, 2 reliable responses should be found in order to allow the possibility of "yes" and "no" for communication.

Responses can be in two different body parts or different parts of the same body part.

If only one reliable response is found, it can be used to answer "yes". E.g. "move your finger if the answer is yes." "Do not move anything if the answer is no".

**Appendix B: Administering the Reuth DOC Response Assessment**

Reuth DOC Response Assessment is comprised of two parts:

1. DOC Movement Checklist- a broad list of movements used to assess the ability to follow commands and/or communicate.
2. DOC Response Profile – the documentation of responses.

**1. DOC Movement Checklist**

First start with observing the spontaneous movements of the patient and then carry out an assessment of the movements as describe in the following guidelines. Finally, assess the movements that were not performed spontaneously.

Indicate on the DOC Movement Checklist table (see below) the following key:

- Indicate "+" for every movement performed according to the checklist (verbal/with clues)
- Indicate "-" for a movement assessed and not performed according to the checklist
- Indicate "?" if there is uncertainty whether the movement was performed as a response to the request/ stimulation or whether the movement was performed spontaneously
- Indicate "**" for an agreed upon movement that symbolizes yes/no pre designated sign or yes/no communication
- Leave the square empty if the movement has not been assessed

Additionally, indicate the type of command instructed using the abbreviations below.

In order to achieve optimal results, the assessor should provide the appropriate setting to allow the movements as indicated in sections 1-5 below. Indicate the type of command instructed on the DOC Movement Checklist table using the abbreviations below. Detailed documenting should be done on the DOC Response Profile indicating in a clear manner the movement performed according to the following guidelines:

Start the assessment using verbal or written instructions

1. Verbal request- (VERB AUD/VERB VIS)
   verbally instruct the patient to perform the movement or present it in written form. If the patient is known or suspected of having a hearing deficit a form of hearing enhancer should be used and information should be presented in written form.
   The instructions should be repeated several times and allow at least 30 seconds for the movement to be performed after the command. If no response is seen to verbal instructions, the assessor should provide the following cues to allow performance of the movement/response. The order of cues is not hierarchical and there is no significance for using one cue over another. If one cue or another does not enable the performance of a movement/response other cues should be attempted.
2. Imitation- (IMIT)
   Demonstrate the desired movement on yourself. Ask the patient to perform the movement while repeating the request.
   1. E.g.-"I'm bending my finger now, you try to bend your finger too".

If

1. Tactile Cue- (TAC)
   Touch the part of the body of the desired movement and request that the patient perform the movement.
   1. E.g. "I'm touching your knee now. Try to move your knee towards my hand /push my hand with your knee". If a reflexive movement appears, wait a few seconds and repeat the instruction.
2. Kinesthetic Cue- (KNST)
   Move the appropriate body part to perform the desired movement and request that the patient repeat the movement.
   1. E.g. "I'm straightening the finger on your right hand. Now you try to straighten it". If a reflexive movement appears, wait a few seconds and repeat the instruction.
3. Giving physical support to perform the desired movement- (SUPP)
   Assess the movement against gravity and in gravity assisted position. Assess with and without the body part supported, eg. with and without a headrest/ in bed. E.g. "I'm holding your arm, try to bring it closer to your body".

**DOC Movement Checklist**

|  |  |  |  |  |  | **Movement / Date** |  |
| --- | --- | --- | --- | --- | --- | --- | --- |
|  |  |  |  |  |  | **Head** | **1** |
|  |  |  |  |  |  | Right |  |
|  |  |  |  |  |  | Left |  |
|  |  |  |  |  |  | Up |  |
|  |  |  |  |  |  | Down |  |
|  |  |  |  |  |  | Nod yes/no |  |
|  |  |  |  |  |  | Full Free movement |  |
|  |  |  |  |  |  | **Eyes** | **2** |
|  |  |  |  |  |  | Opens eyes |  |
|  |  |  |  |  |  | Closes eyes |  |
|  |  |  |  |  |  | Double Blink |  |
|  |  |  |  |  |  | Quick Blink |  |
|  |  |  |  |  |  | Strong Blink |  |
|  |  |  |  |  |  | Prolonged Blink |  |
|  |  |  |  |  |  | Blinks _ times |  |
|  |  |  |  |  |  | Stops Blinking |  |
|  |  |  |  |  |  | Winks |  |
|  |  |  |  |  |  | Full free movement |  |
|  |  |  |  |  |  | **Gaze** | **3** |
|  |  |  |  |  |  | To the right |  |
|  |  |  |  |  |  | To the left |  |
|  |  |  |  |  |  | Up |  |
|  |  |  |  |  |  | Down |  |
|  |  |  |  |  |  | Repeated movements to the sides |  |
|  |  |  |  |  |  | Upon presentation of 2 objects held in the **horizontal** plane, is able to gaze at one of them, on command |  |
|  |  |  |  |  |  | Upon presentation of 2 objects held in the **vertical** plane, is able to gaze at one of them, on command |  |
|  |  |  |  |  |  | Able to gaze at an object/person/picture/word presented beyond the visual field of the patient, on command |  |
|  |  |  |  |  |  | Free full movement |  |
|  |  |  |  |  |  | **Eyebrows** | **4** |
|  |  |  |  |  |  | Contract |  |
|  |  |  |  |  |  | Raise |  |
|  |  |  |  |  |  | Full free movement |  |
|  |  |  |  |  |  | **Mouth** | **5** |
|  |  |  |  |  |  | Open |  |
|  |  |  |  |  |  | Close |  |
|  |  |  |  |  |  | Kiss |  |
|  |  |  |  |  |  | Smile |  |
|  |  |  |  |  |  | A,E,I,O,U vowels |  |
|  |  |  |  |  |  | B,M,P bilabial letters |  |
|  |  |  |  |  |  | D,L,N,T alveolar letters |  |
|  |  |  |  |  |  | Produce sounds |  |
|  |  |  |  |  |  | Full free movement |  |
|  |  |  |  |  |  | **Tongue** | **6** |
|  |  |  |  |  |  | Up |  |
|  |  |  |  |  |  | Down |  |
|  |  |  |  |  |  | Right |  |
|  |  |  |  |  |  | Left |  |
|  |  |  |  |  |  | Stick out |  |
|  |  |  |  |  |  | Move inside the mouth |  |
|  |  |  |  |  |  | Full free movement |  |
|  |  |  |  |  |  | **Upper Extremities** |  |
| L / R | L / R | L / R | L / R | L / R | L / R | **Shoulder** | **7** |
| / | / | / | / | / | / | Elevation |  |
| / | / | / | / | / | / | Flexion |  |
| / | / | / | / | / | / | Extension |  |
| / | / | / | / | / | / | External Rotation |  |
| / | / | / | / | / | / | Internal Rotation |  |
| / | / | / | / | / | / | Full Free movement |  |
| L / R | L / R | L / R | L / R | L / R | L / R | **Elbow** | **8** |
| / | / | / | / | / | / | Flexion |  |
| / | / | / | / | / | / | Extension |  |
| / | / | / | / | / | / | Full Free movement |  |
| L / R | L / R | L / R | L / R | L / R | L / R | **Wrist** | **9** |
| / | / | / | / | / | / | Dorsiflexion |  |
| / | / | / | / | / | / | Plantar flexion |  |
| / | / | / | / | / | / | Ulnar Deviation |  |
| / | / | / | / | / | / | Radial Deviation |  |
| / | / | / | / | / | / | Waves "hello" |  |
| / | / | / | / | / | / | Full Free Movement |  |
|  |  |  |  |  |  | **Hands** | **10** |
| L / R | L / R | L / R | L / R | L / R | L / R | **Thumb** |  |
| / | / | / | / | / | / | Flexion |  |
| / | / | / | / | / | / | Extension |  |
| L / R | L / R | L / R | L / R | L / R | L / R | **Index finger** |  |
| / | / | / | / | / | / | Flexion |  |
| / | / | / | / | / | / | Extension |  |
| L / R | L / R | L / R | L / R | L / R | L / R | **Third finger** |  |
| / | / | / | / | / | / | Flexion |  |
| / | / | / | / | / | / | Extension |  |
| L / R | L / R | L / R | L / R | L / R | L / R | **Fourth Finger** |  |
| / | / | / | / | / | / | Flexion |  |
| / | / | / | / | / | / | Extension |  |
| L / R | L / R | L / R | L / R | L / R | L / R | **Fifth finger** |  |
| / | / | / | / | / | / | Flexion |  |
| / | / | / | / | / | / | Extension |  |
| L / R | L / R | L / R | L / R | L / R | L / R | **Whole Hand** |  |
| / | / | / | / | / | / | Flexion |  |
| / | / | / | / | / | / | Extension |  |
| / | / | / | / | / | / | Grasps an object |  |
| / | / | / | / | / | / | Releases and object |  |
| / | / | / | / | / | / | Points |  |
| / | / | / | / | / | / | Wave "hello" |  |
| / | / | / | / | / | / | Indicates "Yes" |  |
| / | / | / | / | / | / | Indicates "no" |  |
| / | / | / | / | / | / | Full Free Movements |  |
| L / R | L / R | L / R | L / R | L / R | L / R | **Hand shake** |  |
| / | / | / | / | / | / | Once |  |
| / | / | / | / | / | / | Double |  |
| / | / | / | / | / | / | Firm grip |  |
| / | / | / | / | / | / | Brief Grip |  |
| / | / | / | / | / | / | Extended Grip |  |
| / | / | / | / | / | / | Grip _ times |  |
| / | / | / | / | / | / | Releases Grip |  |
| / | / | / | / | / | / | Full Free movement |  |
|  |  |  |  |  |  | **Lower Extremities** |  |
| L / R | L / R | L / R | L / R | L / R | L / R | **Hip** | **11** |
| / | / | / | / | / | / | Flexion |  |
| / | / | / | / | / | / | Extension |  |
| / | / | / | / | / | / | Adduction |  |
| / | / | / | / | / | / | Abduction |  |
| / | / | / | / | / | / | Internal Rotation |  |
| / | / | / | / | / | / | External Rotation |  |
| / | / | / | / | / | / | Full Free Movement |  |
| L / R | L / R | L / R | L / R | L / R | L / R | **Knee** | **12** |
| / | / | / | / | / | / | Flexion |  |
| / | / | / | / | / | / | Extension |  |
| / | / | / | / | / | / | Full Free Movement |  |
| L / R | L / R | L / R | L / R | L / R | L / R | **Foot** | **13** |
| / | / | / | / | / | / | To the Right |  |
| / | / | / | / | / | / | To the Left |  |
| / | / | / | / | / | / | Up |  |
| / | / | / | / | / | / | Down |  |
| / | / | / | / | / | / | Indicates "Yes" |  |
| / | / | / | / | / | / | Indicates "no" |  |
| / | / | / | / | / | / | Full Free Movement |  |
| L / R | L / R | L / R | L / R | L / R | L / R | **Toes** | **14** |
| / | / | / | / | / | / | Flexion |  |
| / | / | / | / | / | / | Extension |  |
| / | / | / | / | / | / | Full Free Movement |  |

**2. DOC Response Profile**

Use the DOC Response Profile (see below) to document the way the movement was performed, in a qualitative manner, with reference to the following:

- Type of instruction
- Communication
- Enhancing conditions
- Restricting Conditions
- Response time
- Consistency
  - During one session
  - During intervention period
- Notes

During the course of the assessment, if movements are observed according to the following clauses, document them in the comments section of the DOC Response Profile table.

- Spontaneously without stimulation
- In response to an unrelated environmental stimulus
- A situational response ( e.g. a smile due to a humorous comment, a kiss towards a family member present)
- A response to a picture/person/object/animal

DOC Response Profile

| Date | Movement/s | Response Profile |
| --- | --- | --- |
|  |  | Command:  Communication:  Enhancing conditions:  Restricting Conditions:  Response time:  Consistency:  - During one session:  - During intervention period:  Notes: |

**Appendix C: Guidelines for the use of the Reuth DOC Periodic Intervention Model**

This model dictates defined times to perform periodic assessment of the patient during the rehabilitation process.
The clinician then uses clinical judgment regarding the continuation, cessation or reinstitution of treatment based on 3 factors:

1. The quality of the response (high level/low level).
2. The consistency of the response/s; how often it appears during a treatment session, and whether the responses are present in each/most/some treatment sessions.
3. Dynamics- is there a change in the number of responses, the level or responses or the consistency of responses.


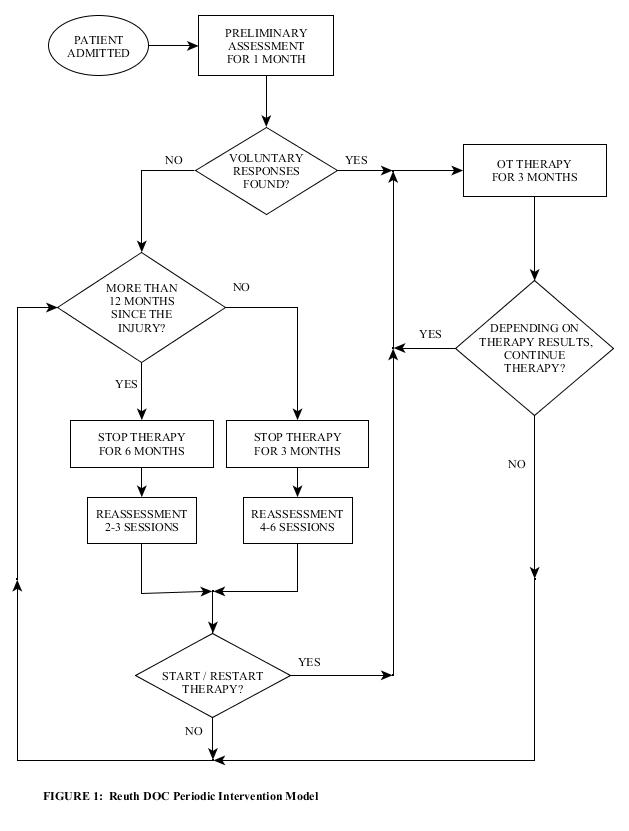

Supplement: Supplementary file 1 [file data_sheet_1.docx]
